# Fellow-Workers and the Roll of Honour

**Published:** 1915-05-08

**Authors:** 


### ROUND THE WORLD.

[The Editor invites Early Information and Contributions Marked "Fellow-Workers."]

### Fellow-Workers and the Roll of Honour.

Among public health officials who have temporarily left their work to undertake military duties must be mentioned Dr. T. B. Heggs, Mr. F. S. Hawks, Dr. W. E. Harker, Mr. H. M. Holt, Dr. A. M. Watts, Dr. J. J. Weaver, Dr. A. Thomson, and Dr. C. C. Finlator.

Dr. Thomas Barrett Heggs, M.D., Ch.B. Aberd., D.P.H. Cantab., studied medicine at the London Hospital as well as at Aberdeen, where he won a scholarship in 1899. Formerly assistant medical officer of health at Brighton, he now holds several important posts in the North-East Kent public health service, including those of medical officer of health and medical inspector of school children; at the same time he is local tuberculosis officer for the county.

A Durham University graduate, Dr. William Edmund Harker qualified in 1893, and subsequently took the degree of D.Hy. as well as the M.D. He was for a time house surgeon to the Royal Infirmary at Newcastle, and is now medical officer of health to the River Tyne Port Sanitary Authority. Some years ago he reported on the sanitary conditions of the River Tyne Port. Having served for awhile on one of the naval hospital ships Dr. Harker has reached the rank of Staff surgeon (R.N.V.R.).

Honorary surgeon to the Malton Cottage Hospital as well as medical officer of health and school medical officer for the district, Mr. Henry Mainwaring Holt has done a good deal of ambulance work in his time, particularly as examiner and lecturer for the St. John Ambulance Association. Formerly a student at Leeds he qualified L.S.A. in 1888, subsequently becoming an M.R.C.S. Eng. and D.P.H. Edin. Mr. Holt was at one time assistant to the professor of physiology at the Yorkshire College of the Victoria University. He has lately been attached to one of the southern military hospitals, whilst one of his sons holds a commission in the 3rd East Lancashires.

Dr. Arthur Thomson, M.B., C.M. Edin., D.P.H. Cantab., occupies a prominent position at Stratford-on-Avon, where he is a member of the staff of the local hospital as well as medical officer of health. After graduating at Edinburgh he travelled for a time as surgeon to one or two shipping companies.

The medical officer of health for Southport, Dr. John James Weaver, who is also on temporary leave for military work, is a University College Hospital man. Qualifying M.R.C.S. Eng., L.S.A. in 1886, and then taking the Cambridge D.P.H., he particularly interested himself in public health problems. For awhile he was house surgeon to the Oldham and Southport Infirmaries, and at Southport now holds also the posts of medical superintendent to the infectious diseases hospital and police surgeon.

Mr. F. S. Hawks, who has joined the R.A.M.C., is one of the Essex County school medical inspectors. A King's College Hospital man, he qualified L.M.S.S.A. in 1908.

An M.D. of Durham University and D.P.H. of Ireland, Dr. Alexander Minter Watts qualified in 1901. On taking up war work he has had to relinquish temporarily the duties of medical officer of health for Ashford and medical inspector of school children for Kent. His past appointments include those of house surgeon at University College Hospital and medical officer of health for Holbeach.

Whilst Dr. T. B. Heggs is away the public health work in North-East Kent will be in the hands of Dr. William McDonald Scott, an Edinburgh graduate, who took the B.Sc degree there in public health, in addition to the M.D. Lately he has been working at Cambridge, where he holds the John Lucas Walker studentship in pathology and a demonstratorship in the same subject. It is understood that Dr. Scott has undertaken special cerebro-spinal fever work for the Government, an assistant having been provided to enable him to continue his administrative duties.

Dr. Cunison Charles Finlator qualified by taking the M.B., Ch.B. at Glasgow in 1903, subsequently proceeding to the M.D. degree, and taking the diploma of D.P.H. of Victoria University. After serving as house surgeon at the Royal Alexandra Infirmary, Paisley, and travelling abroad a good deal as a ship's surgeon, he devoted himself to State medicine, and became appointed to the combined posts of medical officer of health, school medical officer, and tuberculosis officer to the county of Clackmannanshire.

Dr. Ethel Cassie, who has been appointed temporary medical officer of health and school medical officer for Clackmannanshire, in the absence of Dr. Finlator, studied at the Edinburgh School of Medicine for Women and qualified M.B., Ch.B. Edin. in 1906, taking the D.P.H. of Edinburgh and Glasgow in the following year.

Three lady practitioners left England in charge of the Scottish Women's Hospital Unit (2nd Serbian), which sailed on April 20, Dr. Alice M. Hutchison, Dr. A. Louise McIlroy, and Dr. Laura Sandeman. The organiser of the whole foreign service scheme is Dr. Elsie Inglis.

Dr. Alice Marion Hutchison, M.D. Edin., who is in charge of this 2nd Serbian Unit, is physician to the Edinburgh Hospital for Women and Children, and the author of a little work on "Hypnotism and Self-Education." She qualified M.B., Ch.B. Edin., in 1899.

Dr. Annie Louise McIlroy, M.D., Ch.B. Glasgow, chief medical officer of the same unit, is senior assistant to the Muirhead Professor of Obstetrics and Gynæcology at Glasgow University, and assistant gynæcological surgeon at the Glasgow Royal Infirmary. Qualifying in 1898, she subsequently took the L.M. Dublin and D.Sc. Glasgow, in addition to the M.D. degree.

Dr. Elsie Maud Inglis, formerly student of the Edinburgh Medical College for Women and St. Mungo's College, qualified by taking the triple diploma of Edinburgh and Glasgow in 1892, and some years later took the M.B., C.M. at Edinburgh University. Formerly house surgeon to the new Hospital for Women in London, Dr. Inglis now holds at Edinburgh the responsible posts of lecturer on systematic gynæcology in the School of Medicine, joint surgeon and gynæcologist to the Hospital for Women and Children, and obstetric physician to the Hospice.

